# Supplementary material for: Integrating Metabolomics and Transcriptomics to Unveil Atisine Biosynthesis in Aconitum gymnandrum Maxim
Source: Int J Mol Sci. 2022 Nov 3;23(21):13463. doi: 10.3390/ijms232113463 (PMC9655601; doi:10.3390/ijms232113463)
Supplement: Supplementary file 1 [file ijms-23-13463-s001.zip › ijms-1957265-Supplementary.pdf]

International Journal of *Molecular Sciences*

MDPI

Supplementary Material

Integrating Metabolomics and Transcriptomics to Unveil Atisine Biosynthesis in *Aconitum gymnandrum* Maxim

Lingli Chen †, Mei Tian †, Baolong Jin, Biwei Yin, Tong Chen, Juan Guo, Jinfu Tang, Guanghong Cui * and Luqi Huang *

State Key Laboratory Breeding Base of Dao-di Herbs, National Resource Center for Chinese Materia Medica, China Academy of Chinese Medical Sciences, Beijing 100700, China

* Correspondence: cuigh@nrc.ac.cn (G.C.); huanglq@cacms.cn (L.H.)

† These authors contributed equally to this work.

Citation: Chen, L.; Jin, B.; Tian, M.; Yin, B.; Chen, T.; Chun, L.; Guo, J.; Tang, J.; Li, W.; Cui, G.; et al. Integrating Metabolomics and Transcriptomics to Unveil Atisine Biosynthesis in *Aconitum gymnandrum* Maxim. *Int. J. Mol. Sci.* **2022**, *23*, 13463. https://doi.org/10.3390/ijms232113463

Academic Editor: Milen I. Georgiev

Received: 22 September 2022
Accepted: 1 November 2022
Published: 3 November 2022

Publisher's Note: MDPI stays neutral with regard to jurisdictional claims in published maps and institutional affiliations.

Copyright: © 2022 by the authors. Submitted for possible open access publication under the terms and conditions of the Creative Commons Attribution (CC BY) license (https://creativecommons.org/licenses/by/4.0/).

Supplementary Figures

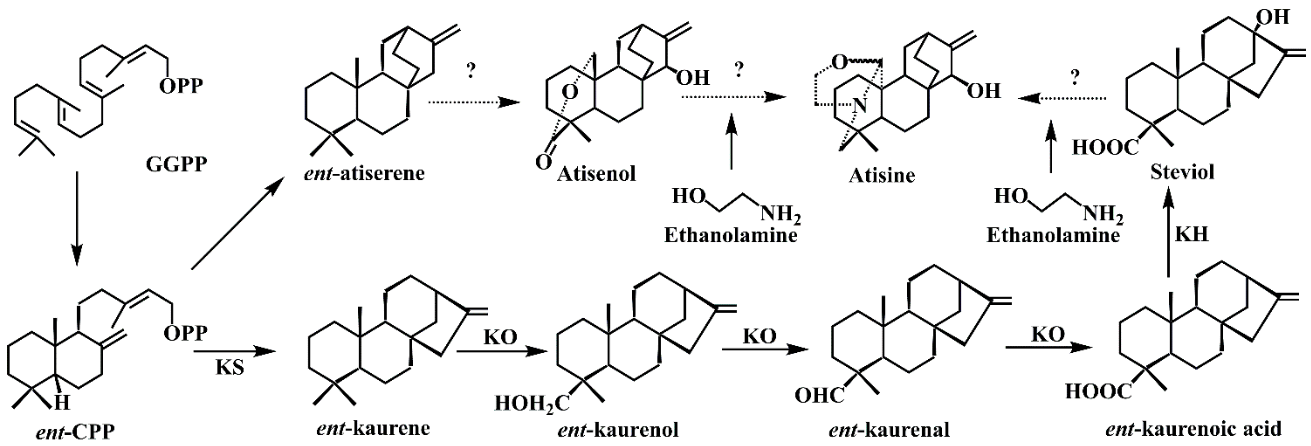

Supplementary Figure S1. Speculative diterpene biosynthetic pathway forming atisine in *A. heterophyllum*. Question marks indicate missing enzymes.

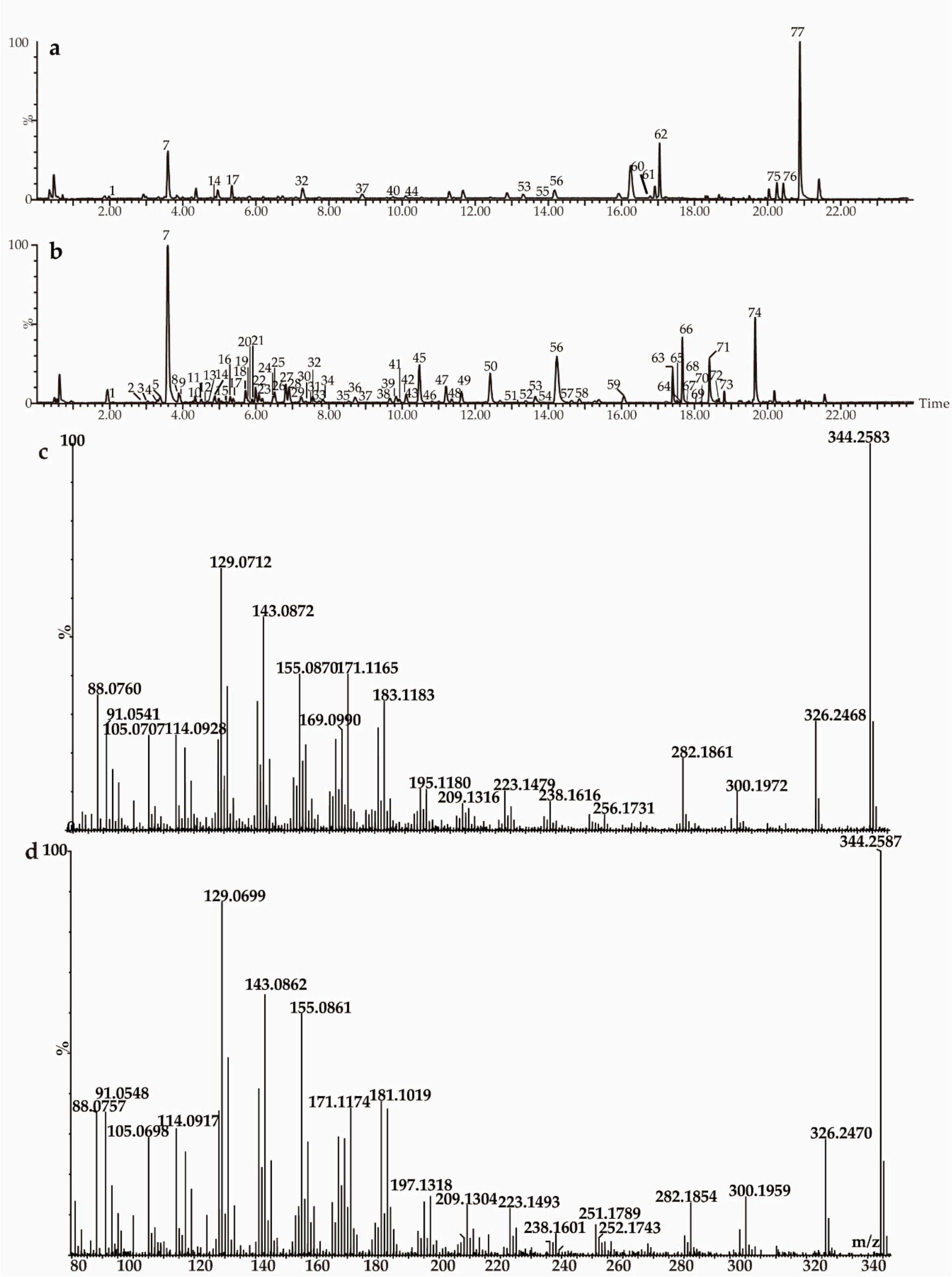

Supplementary Figure S2. The base peak ion diagram of *A. gymnandrum*, the peak number is the same as that in TabS1. (**a**) Chromatogram of aerial portions; (**b**) Chromatogram of root;(**c**) Mass spectrum of atisine reference substance; (**d**) Mass spectrum of atisine in the sample group.

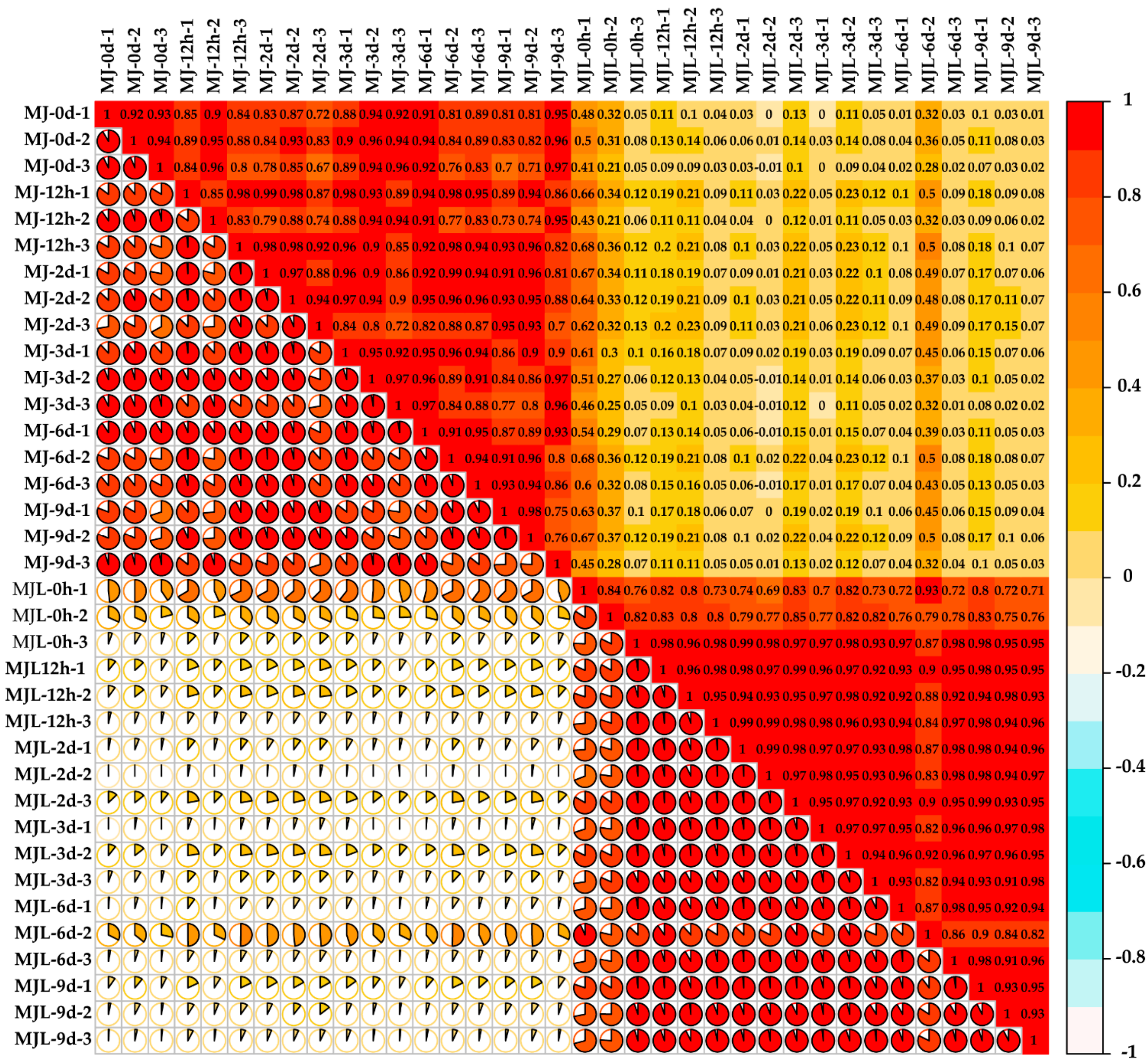

Supplementary Figure S3. Correlation of samples with different induction time.

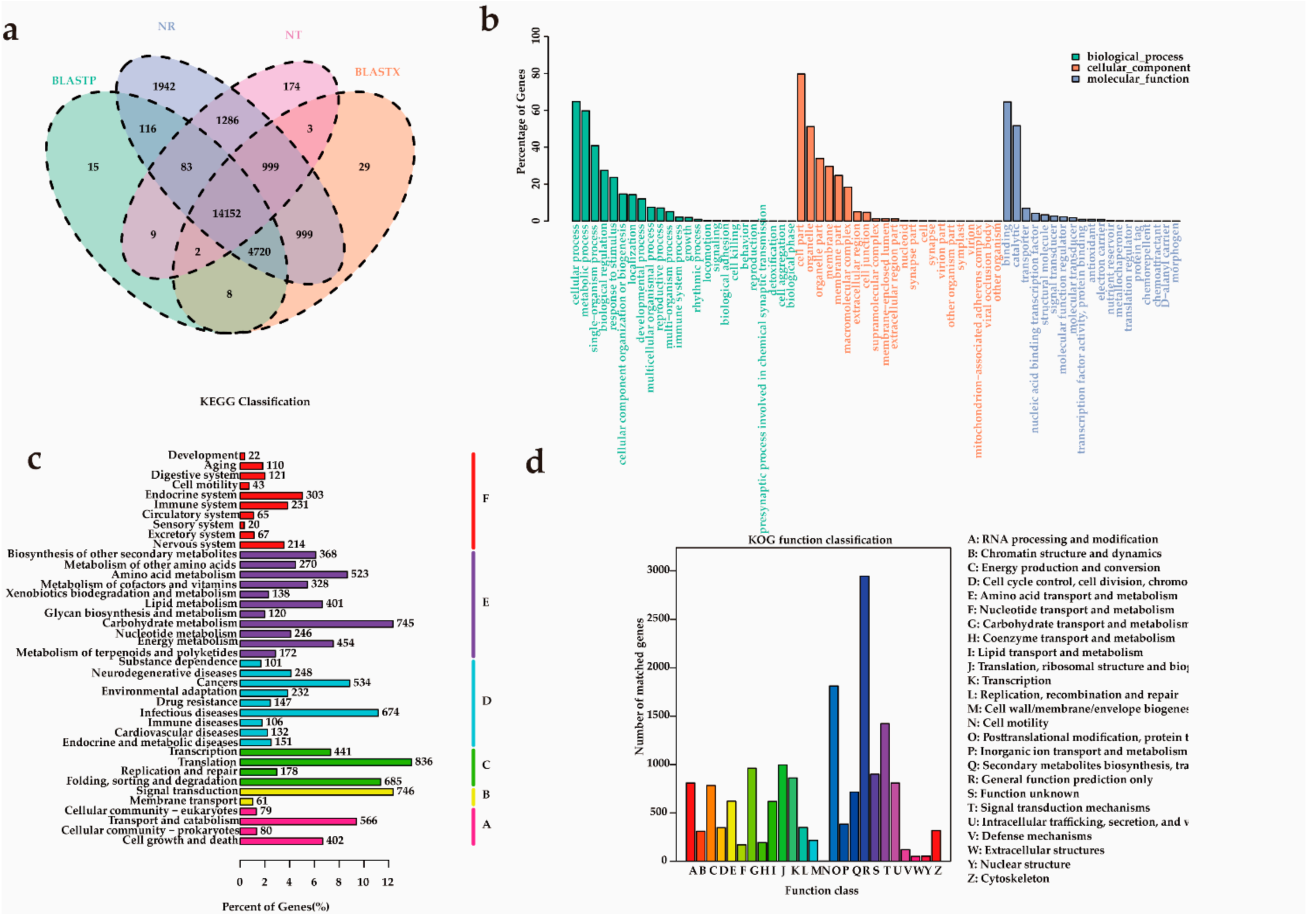

Supplementary Figure S4. Annotation and classification of genes in public databases in *A. gymnandrum* (**a**) Comparison of annotation results in NT, NR, blasts, and blast databases venn diagram; (**b**) Functional annotation of genes based on GO categorization, The main functional categories within the biological process, cellular component and molecular function categories; (**c**) KEGG annotation classification statistical chart. A: Cellular Processes, B: Environmental Information Processing, C: Genetic Information Processing, D: Human Diseases, E: Metabolism, F: Organismal Systems (**d**) statistical chart of KOG, t the abscissa is the category in the KOG database, and the ordinate is the number of matched genes. the number of matched genes.

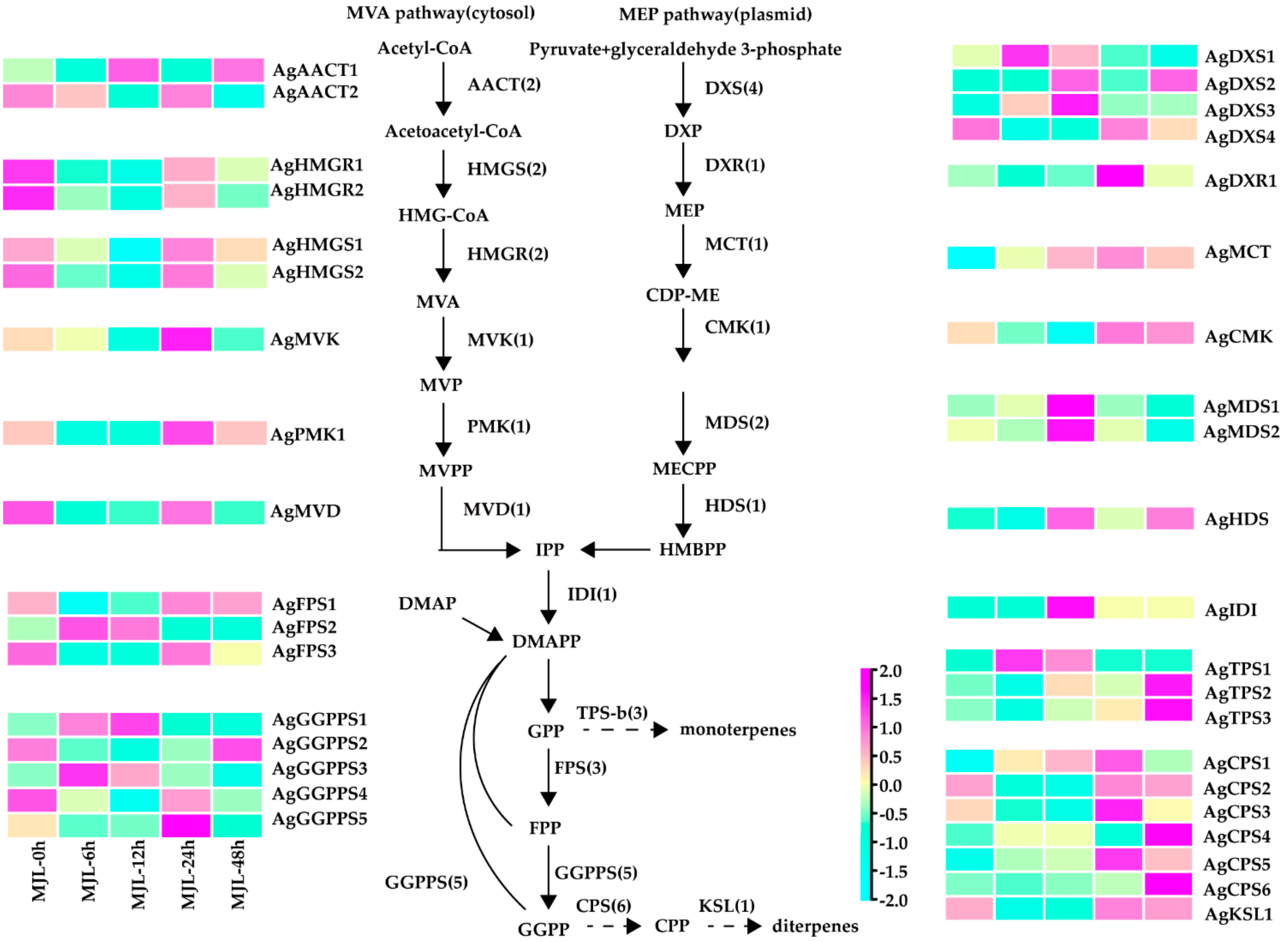

Supplementary Figure S5. The expression profile for the genes involved in terpenoid biosynthesis in the aerial portions of *A. gymnandrum*. The expression of each identified gene is represented as the FPKM after Log2 standardization of *A. gymnandrum* transcriptomes. Abbreviations: Abbreviations: AACT, aceto-acetyl-CoA thiolase; CMK, 4-(cytidine 50 -diphospho)-2-C-methyl-D-erythritol kinase; DXS, 1-deoxy-D-xylulose 5-phosphate synthase; DXR, 1-deoxy-D-xylulose-5-phosphate reductoisomerase; FPS, Farnesyl pyrophosphate synthase; HDR, (E)-4-hydroxy-3-methylbut-2-enyl diphosphate reductase; HDS, (E)-4-hydroxy-3-methylbut-2-enyl diphosphate synthase; HMGS, 3-hydroxy-3-methylglutaryl-CoA synthase; HMGR, 3-hydroxy-3-methylglutaryl-CoA reductase; GGPPS, Geranylgeranyl pyrophosphate synthase; MCT, 2-C-methyl-D-erythritol 4-phosphate cytidylyltransferase; IDI, Isopentenyl diphosphate isomerase; MDS, 2-C-methyl-D-erythritol 2,4-cyclodiphosphate synthase; MDD, Mevalonate diphosphate decarboxylase; MVK, mevalonate kinase; PMK, phosphomevalonate kinase; TPS, terpene synthases (including monoterpene syn-thases and sesquiterpene synthases); CPS, copalyl diphosphate synthase; KSL, Kaurene synthase.

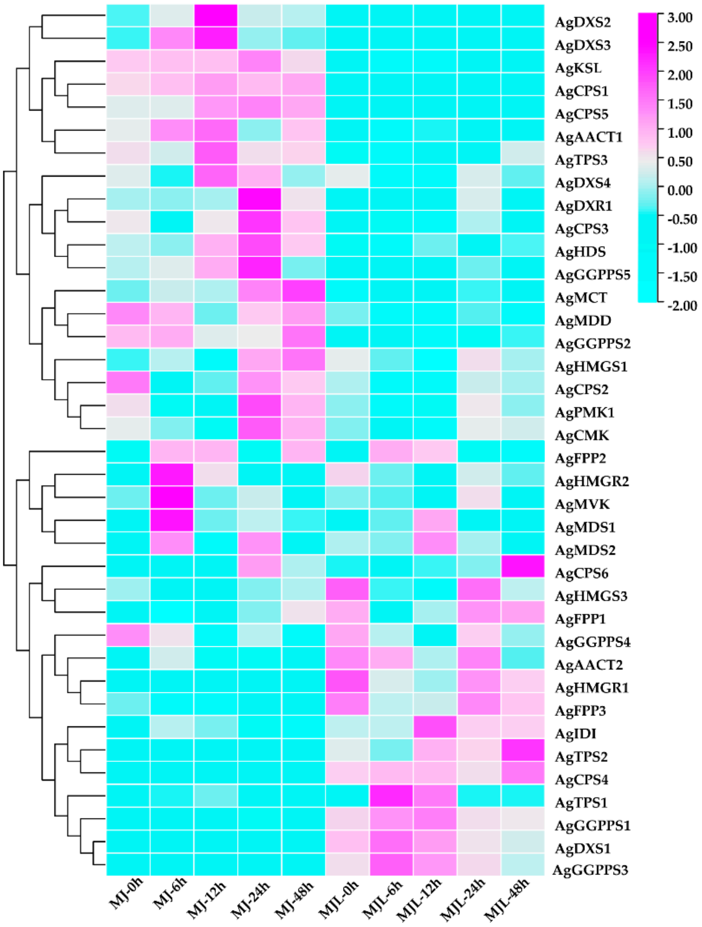

Supplementary Figure S6. Heat map of MVP pathways, MEP pathways, and terpene synthase gene average FPKM (row scale) in roots and aerial portions of *A. gymnandrum*.

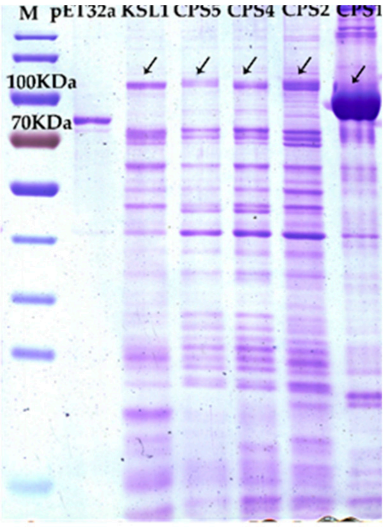

Supplementary Figure S7. SDS-PAGE electrophoresis of AgKSL and AgCPSs crude proteins (M: marker).

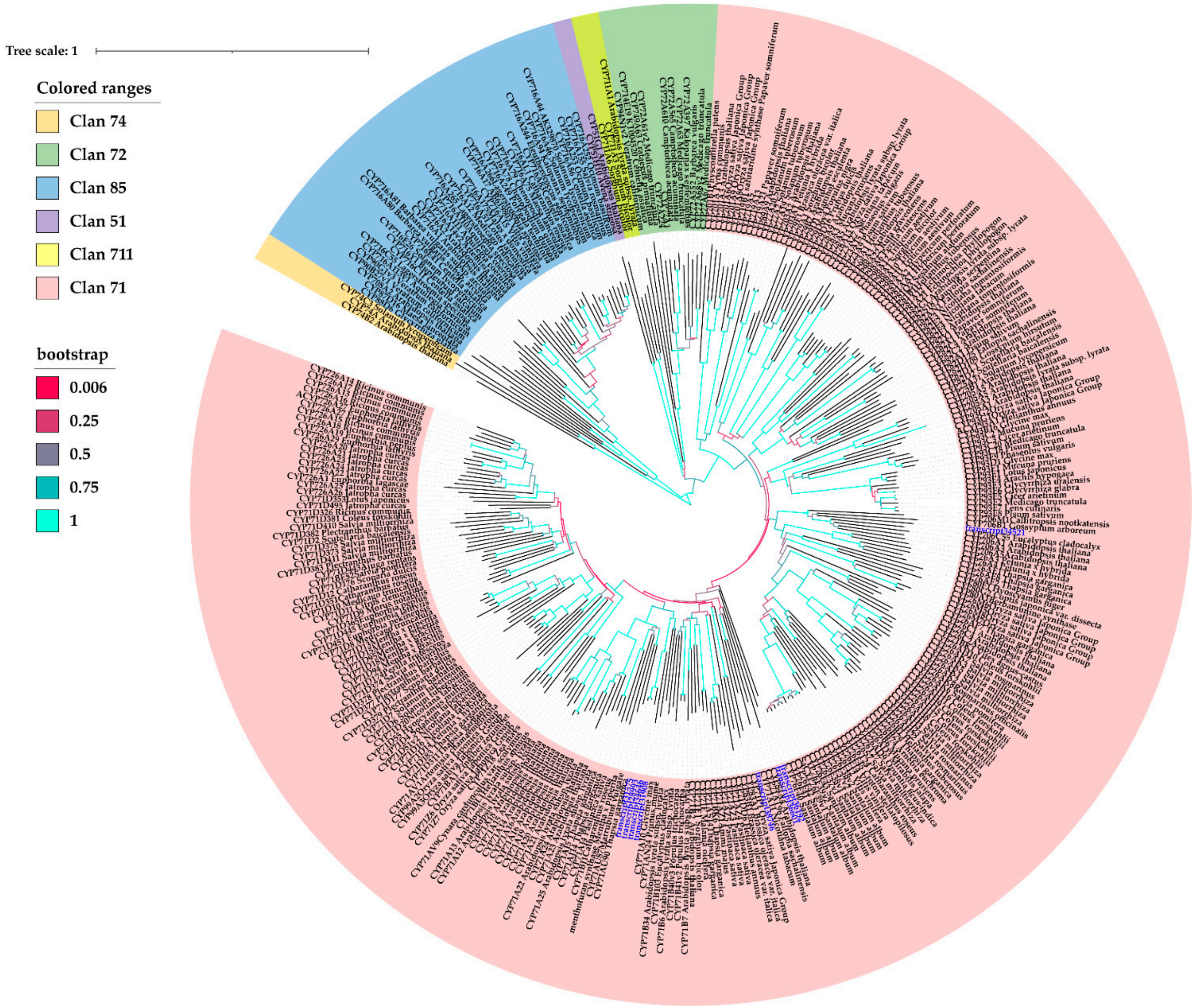

Supplementary Figure S8. Phylogenetic analysis of CYP450 in the *A. gymnandrum*. The blue words indicate the CYP450 genes screened by WGCNA analysis. All sequences were downloaded from NCBI or Unipro.

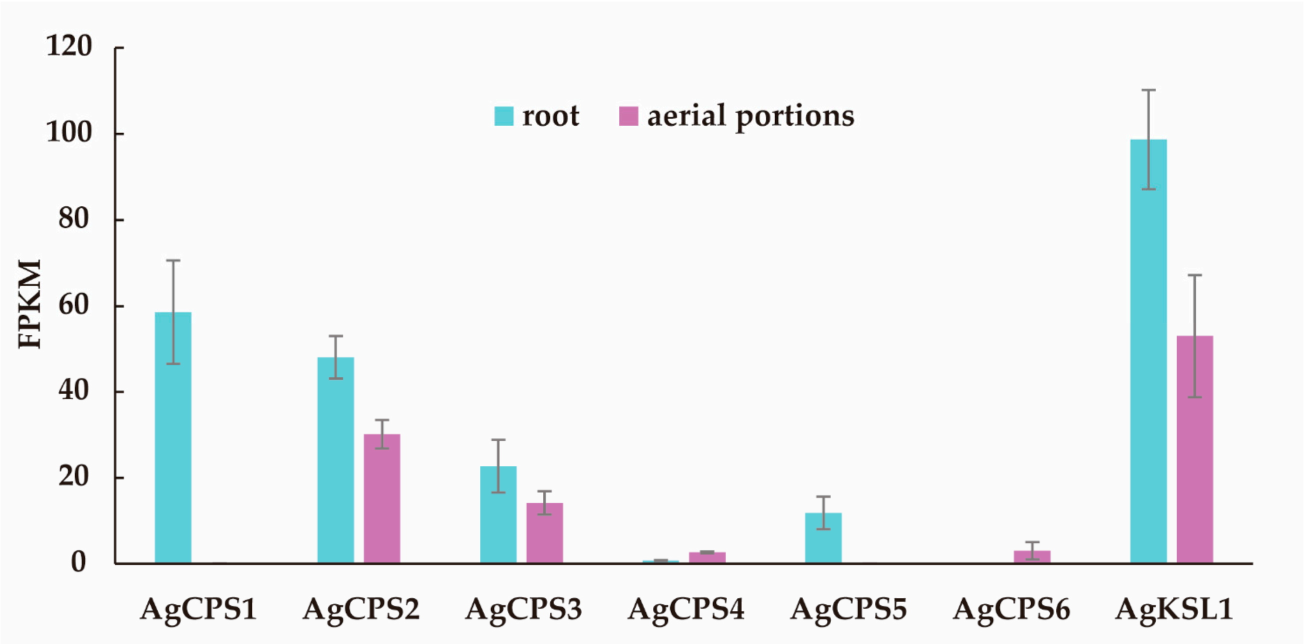

Supplementary Figure S9. The expression level of diterpene synthase genes in root and aerial portions of A. gymnandrum.

Supplementary Table S7. Primer sequences of AgCPS and AgKSL genes used in functional identification.

Genes	Primer sequences
AgCPS1-BamH1-F	gccatggctgatatcggatccATGGCTTCTCTCTACACCTCCCTC
AgCPS1-Sal1-R	gcaagcttgtcgacggatccTTAAGCTACTCTCTTAAAGAGGACATCAG
AgCPS2-BamH1-F	ggccatggctgatatcggatccATGTCTTCTCTCTCTTCTTCCCTC
AgCPS2-Sal1-R	cggccgcaagcttgtcgacTTAGCCTACTCTCTTAAATAAAACTTCAG
AgCPS3-BamH1-F	ggccatggctgatatcggatccATGTCTTCTCTCTCTTCTACTCTCAG
AgCPS3-Sal1-R	gccgcaagcttgtcgacTTAGTTCACTCTCTTAAACAACACTTGAG
AgCPS4-BamH1-F	ggccatggctgatatcggatccATGTCTTCTCTCTCTTCTACTCTCAG
AgCPS4-Sal1-R	ggccgcaagcttgtcgacTCAGTTCATTCTCTTAAACAACACTTCG
AgCPS5-BamH1-F	ggccatggctgatatcggatccATGTCTTTTGTGCTTAATCTTCTCCC
AgCPS5-Sal1-R	ggccgcaagcttgtcgacTCAAATAACTGGATCAAATAGAACTTTGCC
AgKSL1-BamH1-F	ggccatggctgatatcggatccATGTATCTCTCTCACCCAAACAAGTC
AgKSL1-Sal1-R	gccgcaagcttgtcgacCTAGCCTTCAGTTAAATCTGGTCTAGTG
